# RETRACTION: Effect of Cefuroxime Intracameral Injection Antibiotic Prophylactic on Postoperative Endophthalmitis Wound Post‐Cataract: A Meta‐Analysis

**DOI:** 10.1111/iwj.70557

**Published:** 2025-04-18

## Abstract

**RETRACTION:** YuL. , LiL. , LiD. , LuoZ. , LiuN. , WuY. , BaoD. , and CuiX. , “Surgical Strategy of Lumbopelvic Instrumentation in the Treatment of Lumbosacral Tuberculosis: S2‐Alar‐Iliac Screws vs Iliac Screws,” International Wound Journal 19, no. 8 (2022): 1964–1974, 10.1111/iwj.13795.35297177 PMC9705182

The above article, published online on 16 March 2022, in Wiley Online Library (http://onlinelibrary.wiley.com/), has been retracted by agreement between the journal Editor in Chief, Professor Keith Harding; and John Wiley & Sons Ltd. Following an investigation by the publisher, all parties have concluded that this article was accepted solely on the basis of a compromised peer review process. The editors have therefore decided to retract the article. The authors did not respond to our notice regarding the retraction.

**RETRACTION:** L. Yu , L. Li , D. Li , Z. Luo , N. Liu , Y. Wu , D. Bao , and X. Cui , “Surgical Strategy of Lumbopelvic Instrumentation in the Treatment of Lumbosacral Tuberculosis: S2‐Alar‐Iliac Screws vs Iliac Screws,” International Wound Journal 19, no. 8 (2022): 1964–1974, 10.1111/iwj.13795.35297177 PMC9705182

The above article, published online on 16 March 2022, in Wiley Online Library (http://onlinelibrary.wiley.com/), has been retracted by agreement between the journal Editor in Chief, Professor Keith Harding; and John Wiley & Sons Ltd. Following an investigation by the publisher, all parties have concluded that this article was accepted solely on the basis of a compromised peer review process. The editors have therefore decided to retract the article. The authors did not respond to our notice regarding the retraction.

